# A Stable Chimeric Fibroblast Growth Factor (FGF) Can Successfully Replace Basic FGF in Human Pluripotent Stem Cell Culture

**DOI:** 10.1371/journal.pone.0118931

**Published:** 2015-04-07

**Authors:** Yasuko Onuma, Kumiko Higuchi, Yasuhiko Aiki, Yujing Shu, Masahiro Asada, Makoto Asashima, Masashi Suzuki, Toru Imamura, Yuzuru Ito

**Affiliations:** 1 Research Center for Stem Cell Engineering, National Institute of Advanced Industrial Science and Technology (AIST), Tsukuba Central 4, 1-1-1 Higashi, Tsukuba, Ibaraki 305–8562, Japan; 2 Signaling Molecules Research Group, Biomedical Research Institute, National Institute of Advanced Industrial Science and Technology (AIST), Tsukuba Central 6, 1-1-1 Higashi, Tsukuba, Ibaraki 305–8566, Japan; 3 Graduate School of Science and Engineering, Ibaraki University, 2-1-1 Bunkyo, Mito, Ibaraki 310–8512, Japan; 4 Cell Regulation Laboratory, School of Bioscience and Biotechnology, Tokyo University of Technology, 1404–1 Katakura Hachioji, Tokyo 192–0982, Japan; IDI, Istituto Dermopatico dell'Immacolata, ITALY

## Abstract

Fibroblast growth factors (FGFs) are essential for maintaining self-renewal in human embryonic stem cells and induced pluripotent stem cells. Recombinant basic FGF (bFGF or FGF2) is conventionally used to culture pluripotent stem cells; however, because of the instability of bFGF, repeated addition of fresh bFGF into the culture medium is required in order to maintain its concentration. In this study, we demonstrate that a heat-stable chimeric variant of FGF, termed FGFC, can be successfully used for maintaining human pluripotent stem cells. FGFC is a chimeric protein composed of human FGF1 and FGF2 domains that exhibits higher thermal stability and protease resistance than do both FGF1 and FGF2. Both human embryonic stem cells and induced pluripotent stem cells were maintained in ordinary culture medium containing FGFC instead of FGF2. Comparison of cells grown in FGFC with those grown in conventional FGF2 media showed no significant differences in terms of the expression of pluripotency markers, global gene expression, karyotype, or differentiation potential in the three germ lineages. We therefore propose that FGFC may be an effective alternative to FGF2, for maintenance of human pluripotent stem cells.

## Introduction

Basic fibroblast growth factor (bFGF, also known as FGF2) is an essential exogenous growth factor required for maintaining the self-renewal of human embryonic stem cells (hESCs) and induced pluripotent stem cells (hiPSCs) [1–5]. bFGF activates mitogen-activated protein kinase (MAPK) kinase (MEK)/extracellular signal-regulated kinase (ERK) and phosphoinositol 3-kinase (PI3K)/AKT pathways via FGF receptors (FGFRs) and maintains hESCs/iPSCs in an undifferentiated state [6–9]. Given the importance of FGF signaling, rapid loss of bFGF protein in culture medium (by more than 50% in 4 h) due to its vulnerability to heat and proteases, is a serious problem in maintaining the quality of cultures [10]. A recent study on the development of beads that enable sustained levels of bFGF in the culture media highlights the need for solutions to this problem [10].

We have previously reported the development of FGFC, a chimeric protein consisting of FGF1 and FGF2 fragments that is thermally and proteolytically stable and does not require heparin to activate FGF receptors [11]. In this study, we demonstrate the potential for FGFC to replace FGF2 as a growth factor used in the maintenance of pluripotent hESCs and hiPSCs. FGFC activated the phosphorylation of ERK1, ERK2, and p38γ in 15 min, similar to the activation of these pathways by bFGF in hESCs. We also analyzed hESCs after a long-term (more than 30 days) culture in FGFC-containing medium in comparison with those cultured with bFGF: The hESCs grown in FGFC media, did not show any significant differences in the expression of pluripotency markers, global gene expression, karyotype, or differentiation potential into the three germ lineages. Similar results were obtained in hiPSCs. Together, these results suggest FGFC as a functional and convenient alternative to bFGF that holds promise in improving cell culture methods for human ESCs and iPSCs

## Materials and Methods

### Ethics statement

This study was carried out in strict accordance with the National Institute of Advanced Industrial Science and Technology (AIST) guidelines for life science experiments, and all human pluripotent stem cell experiments were approved by the Ministry of Education, Culture, Sports, Science, and Technology of Japan (MEXT; accreditation numbers 2013–099 and 2013–078).

### Preparation of recombinant FGFC protein

Recombinant FGFC was expressed in a prokaryotic expression system, as described previously [11]. BL21(DE3)pLysS *Escherichia coli* cells were transformed with the FGFC/pET3c vector and were then propagated in LB medium using an Overnight Express Autoinduction System 1 (Novagen). The expressed FGFC protein was extracted and purified on a Heparin-Sepharose column (Amersham) by washing with 0.7 M NaCl, 20 mM Tris–HCl (pH 7.4) and eluting with 1.5 M NaCl in 20 mM Tris-HCl (pH 7.4). The protein was then further purified on a Hi-Trap heparin HPLC column by using a linear NaCl gradient in 20 mM Tris-HCl (pH 7.4). The obtained protein was shown to be pure by Coomassie Brilliant Blue staining after sodium dodecyl sulfate-polyacrylamide gel electrophoresis. Purified FGFC protein was stored at -80°C until further use [11].

### Cell culture

The human ES cell line H1 [12] was maintained in mTeSR1 (STEMCELL Technologies) on the BD Matrigel Growth Factor Reduced (GFR) matrix (BD Biosciences), according to WiCell Feeder Independent Pluripotent Stem Cell Protocols provided by the WiCell Research Institute (www wicell.org). H1 cells were also cultured in E8 Medium (Essential 6 Medium [Life technologies] plus 2 ng/mL of TGF-beta 1 [R&D Systems] with 100 ng/mL of bFGF [PeproTech]) on a BD Matrigel GFR matrix (BD Biosciences), according to WiCell Feeder-Independent Pluripotent Stem Cell Protocols E8 Medium provided by the WiCell Research Institute (www.wicell.org).

Mitomycin C-treated mouse embryonic fibroblast (MMC-MEF) conditioned medium was prepared according to the Human Pluripotent Stem Cell Protocols provided by the Human Stem Cell Technology Unit, RIKEN Center for Developmental Biology (www.cdb.riken.jp/hsct/protocol.html). H1 cells were cultured with the MEF-conditioned medium on BD Matrigel GFR (BD Biosciences).

The human ES cell line KhES-1 [13] was maintained as previously described [13]. The human iPS cell line 201B7 [14] was cultured in DMEM-F12 medium (Invitrogen) supplemented with 20% KnockOut Serum Replacement (KSR; Invitrogen), 0.1 mM of 2-mercaptoethanol (Sigma-Aldrich), MEM Non-essential Amino Acids (Invitrogen), and 10 ng/mL of recombinant human basic FGF (Wako) on MMC-MEF as feeder cells. The human iPS cell line 253G1 [15] was cultured in E8 Medium on a BD Matrigel matrix (BD Biosciences).

To investigate the efficacy of FGFC, hESCs and hiPSCs were cultured with maintenance medium containing FGFC instead of bFGF. The concentration of FGFC was the same as the standard bFGF concentration used for culture of these cells (5–100 ng/mL, depending on the culture medium). G-banded karyotyping was performed at Nihon Gene Research Laboratories Inc. (Japan).

### Signal pathway analysis

A Proteome Profiler Human Phospho-MAPK Array Kit (R&D Systems) was used for analysis of the relative levels of phosphorylation of mitogen-activated protein kinases (MAPKs) and other serine/threonine kinases induced by FGFC treatment (S1 Table). H1 ESCs were cultured in Essential 6 Medium (Life technologies) plus 2 ng/mL of TGF-beta 1 (R&D Systems) without bFGF for 4 days, then were treated with either 100 ng/mL of bFGF or 100 ng/mL of FGFC for 15 min. Protein extraction and further analysis were performed according to the manufacturer’s instructions (R&D Systems).

### *In vitro* differentiation assays

H1 ESCs were cultured in E8 Medium or E8 Medium with 100 ng/mL of FGFC, instead of bFGF, for 30 days, through 10 passages. Cell colonies were detached by treatment with 0.25% trypsin (Life Technologies), 1 mg/mL collagenase type IV (Gibco), 20% KSR (Invitrogen), and 1 mM CaCl_2_ in phosphate-buffered saline (PBS), and were transferred into a HydroCell dish (CellSeed Inc.) in DMEM-F12 medium (Invitrogen) supplemented with 20% KSR (Invitrogen), 0.1 mM of 2-mercaptoethanol (Sigma-Aldrich), and MEM non-essential amino acids (Invitrogen) for embryoid body (EB) formation. The medium was changed every other day. EBs were attached to a gelatin-coated multi-well plate at day 8 and cultured in the same medium for another 11 days for immunocytochemistry analysis.

### Immunocytochemistry and lectin staining

Immunocytochemical analysis was performed as described previously [16–18]. The following primary antibodies were used in this study: anti-SSEA4 (1:300 dilution; Millipore), anti-TRA-1-60 (1:300 dilution; Millipore), anti-TRA-1-81 (1:300 dilution; Millipore), anti-Oct-3/4 (1:300 dilution; Santa Cruz Biotechnology), anti-human Nanog (1:800 dilution; Cell Signaling Technology), anti-Class IIIβ-tubulin (TUJ1; 1:500; Covance), anti-human smooth muscle actin (SMA; 1:50; DAKO), and anti-α-fetoprotein (1:100; R&D systems). Cells were incubated with a primary antibody diluted in 1% bovine serum albumin (BSA) and 5% serum containing PBS at 4°C overnight. Secondary staining was performed with an appropriate secondary antibody-conjugated to Alexa Fluor 488 or Alexa Fluor 594 (1:300; Life technologies) for 1 h at room temperature. Lectin staining was performed as described previously [19,20]. Briefly, rBC2LCN (Wako) was fluorescent-labeled using FITC Labeling Kit-NH2 (Dojindo) according to the manufacturer’s instruction. Next, 10 μg/mL of FITC-conjugated rBC2LCN in 1% BSA containing PBS was used for staining of 4% paraformaldehyde-fixed cells for 1 h at room temperature. Cells were counterstained with DAPI (Dojindo). Images were collected with a BIOREVO BZ-9000 fluorescence microscope (Keyence).

### DNA microarray and analysis

Total RNA was extracted from frozen cell samples using ISOGEN (NIPPON GENE) according to the manufacturer’s instructions. Samples were analyzed using an Agilent SurePrint G3 Human GE 8 × 60K Microarray Kit (G4851A) and a one-color Low Input Quick Amp Labeling Kit (Agilent). Arrays were scanned using a G2505C Microarray Scanner System (Agilent). Raw microarray data were submitted to the Gene Expression Omnibus (GEO) microarray data archive (http://www.ncbi.nlm.nih.gov/geo/) at NCBI (accession number GSE55428).

Data were analyzed using Gene-Spring GX12.0 software (Agilent) after applying two normalization procedures. First, signal intensities of less than 1 were set to 1, and then each chip was normalized to the 75th percentile of all measurements from that chip. Baseline transformation of these data was not performed. Genes with a flag value of “detected” or “not detected” in at least one sample were analyzed. Differentially expressed genes were selected if the average values of each sample were altered by at least 2.0-fold. Early differentiation marker genes for the heat map were selected as previously described [21]. GO terms enriched with a p-value cut-off of 0.01 were extracted.

### Quantitative real-time polymerase chain reaction (PCR) analysis

Total RNA was extracted from frozen cell samples using ISOGEN (NIPPON GENE) according to the manufacturer’s instructions. One-step real-time PCR analysis was carried out using a One Step SYBR PrimeScript PLUS RT-PCR Kit (Takara Bio Inc.) with Chromo4 and DNA Engine Opticon 2 real-time PCR detection systems (Bio-Rad) using the following program: 42°C for 5 min, 95°C for 10 s, and then 35 cycles of 94°C for 15 s, 60°C for 30 s, and 72°C for 30 s, with each cycle followed by a plate read. Ten nanograms of total RNA template and 0.4 μM of each primer were used for the reaction. The primer sequences were published previously [22], with the exception of primers for *Dnmt3b* and *Rex1*, which were modified for the detection of accurate human transcripts as follows: *Dnmt3b* (NM_006892), sense GCTAAGCTACACACAGGACTTGACAG and antisense AGTTCGGACAGCTGGGCTTT; *Rex1* (NM_174900), sense CAGATCCTAAACAGCTCGCAGAAT and antisense GCGTACGCAAATTAAAGTCCAGA (modified regions are underlined). Each reaction was performed in triplicate, and all results were normalized to *Gapdh* expression.

## Results and Discussion

### FGFC activated similar intracellular signals similar to those induced by bFGF in human embryonic stem cells

**Fig 1:**
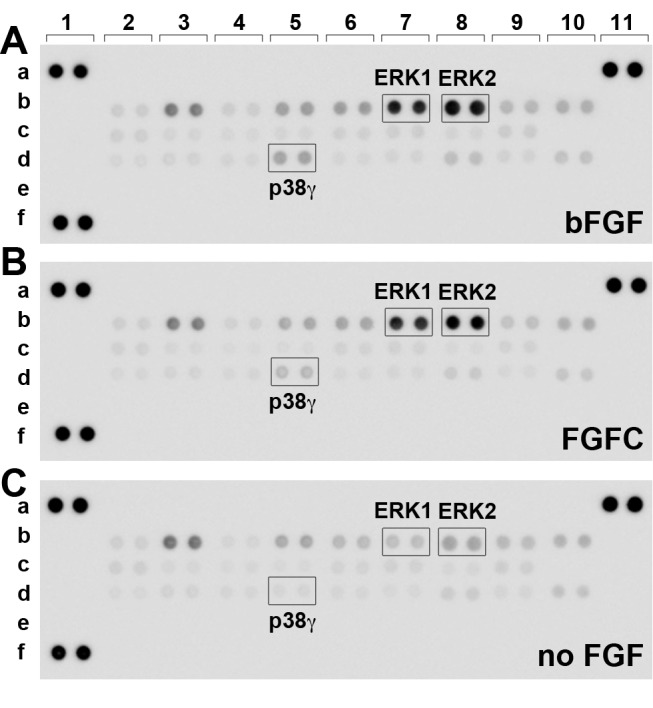
FGFC induced phosphorylation of MAPKs in human ESCs. H1 hESCs were treated with E8 Medium with either 100 ng/mL bFGF (A), 100 ng/mL FGFC (B), or no added FGF (C) for 15 min. Each antibody was spotted in duplicate. A typical data set from two independent experiments is shown. b2: Akt1, b3: Akt2, b4: Akt3, b5: Akt pan, b7: ERK1, b8: ERK2, b9: GSK-3α/β, b10: GSK-3β, d3: p38δ. Array coordinate details are given in S1 Table.

First, we examined whether FGFC activated the same intracellular signals as bFGF in hESCs. To compare the induction of signaling pathways in hESCs by stimulation with FGFC versus bFGF, we used a Proteome Profiler Human Phospho-MAPK Array to simultaneously detect the relative phosphorylation levels of 26 kinases, including nine MAPKs (S1 Table). The overall phosphorylation profile of FGFC-cultured cells was similar to that of bFGF-cultured cells and was different from that of control cells without FGF treatment (Fig 1). The phosphorylation levels of ERK1 and ERK2, and p38γ were increased under both bFGF and FGFC treatment. AKT and glycogen synthase kinase 3 β (GSK3β) have been reported to be regulated by the FGF signal [8]. In our study, however, these proteins did not exhibit significant increases in phosphorylation under either bFGF or FGFC treatment, but exhibited marked increases in phosphorylation in the absence of FGF (Fig 1C, AKT; b2-b5, b9-10; GSK-3β). This may be explained by the observation that AKT and GSK3β are downstream of the insulin/PI3-K pathway, as well as the FGF pathway. Thus, the induction of signaling pathways via the insulin present in the Essential 6 Medium may mask any induced increases caused by FGFs. These results indicate that FGFC induced similar to those induced by bFGF in hESCs.

### FGFC sustained global gene expression in human pluripotent stem cells

**Fig 2:**
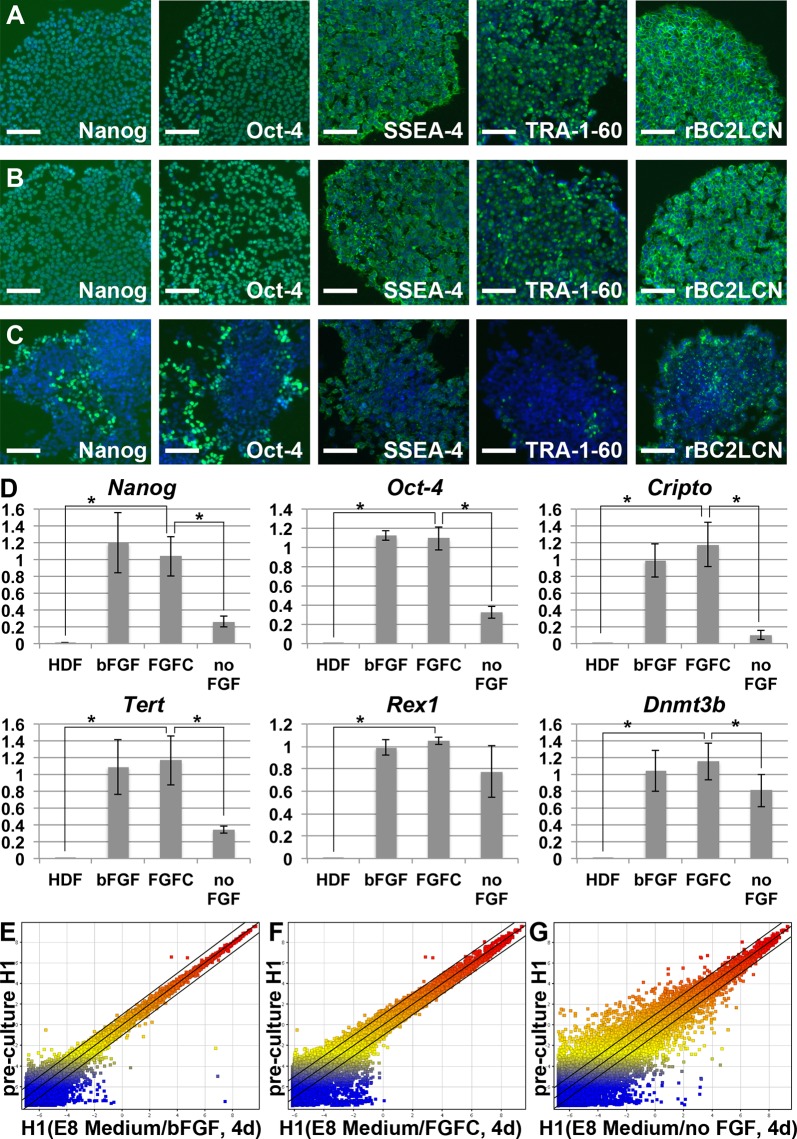
FGFC did not affect the expression of pluripotent markers in human ESCs. (A-D) Pluripotent marker expression in H1 ESCs cultured in E8 Medium with bFGF (A), FGFC (B), or no added FGF (C) for 4 days. A typical data set from two independent experiments is shown. (D) Expression levels of pluripotent marker genes (*Nanog*, *Oct-4*, *Cripto*, *Tert*, *Rex1* and *Dnmt3b*) were analyzed by quantitative RT-PCR. The graphs represent relative gene expression levels when the level of H1 ESCs prior to starting each series of experiments was set as 1. Error bars indicate the standard deviations of three independent experiments. Human dermal fibroblasts (HDFs) were used as a negative control. *, P < 0.05 compared to ESCs cultured with FGFC by paired t-tests. (E–G) Scatter plots showing log_2_ transformed average expression values from gene expression profiles of H1 ESCs prior to starting a series of cultures (pre-culture H1) and H1 ESCs cultured with bFGF (E), FGFC (F), or no FGF (G) using arrayed 60 k probe sets. A typical data set from three independent experiments is shown. The full array data set was deposited in the GEO databank (GSE55428). Black lines indicate 2-fold up-regulation or down-regulation. Scale bar: 100 μm in (A-C).

We next investigated whether human pluripotent stem cells were stably maintained in FGFC-containing medium (Fig 2). H1 ESCs were cultured in E8 Medium containing 100 ng/mL of FGFC, instead of bFGF, for 4 days. Immunostaining for Nanog, Oct-4, SSEA-4, Tra-1-60, and lectin staining of rBC2LCN, indicated that FGFC-treated hESCs maintained their undifferentiated state as reliably as bFGF-treated cells (Fig 2A and 2B). At the start of differentiation, hESCs exhibited decreases in the expression levels of pluripotency markers in the absence of FGF (Fig 2C). The expression levels of pluripotent cell marker genes (i.e., *Nanog*, *Oct-4*, *Cripto*, *Tert*, *Rex1*, and *Dnmt3b*) did not differ significantly between H1 ESCs cultured with bFGF and those cultured with FGFC in quantitative RT-PCR analysis (Fig 2D). We further analyzed the global gene expression of H1 hESCs cultured in FGFC-containing medium, using a DNA microarray (Fig 2E–2G). The scatter plots showed very similar gene expression profiles in bFGF- and FGFC-treated cells. These results suggested that FGFC induced and maintained a gene expression profile similar to that of hESCs cultured in bFGF-containing media.

### Long-term replacement with FGFC maintained the pluripotency of human ES cells

**Fig 3:**
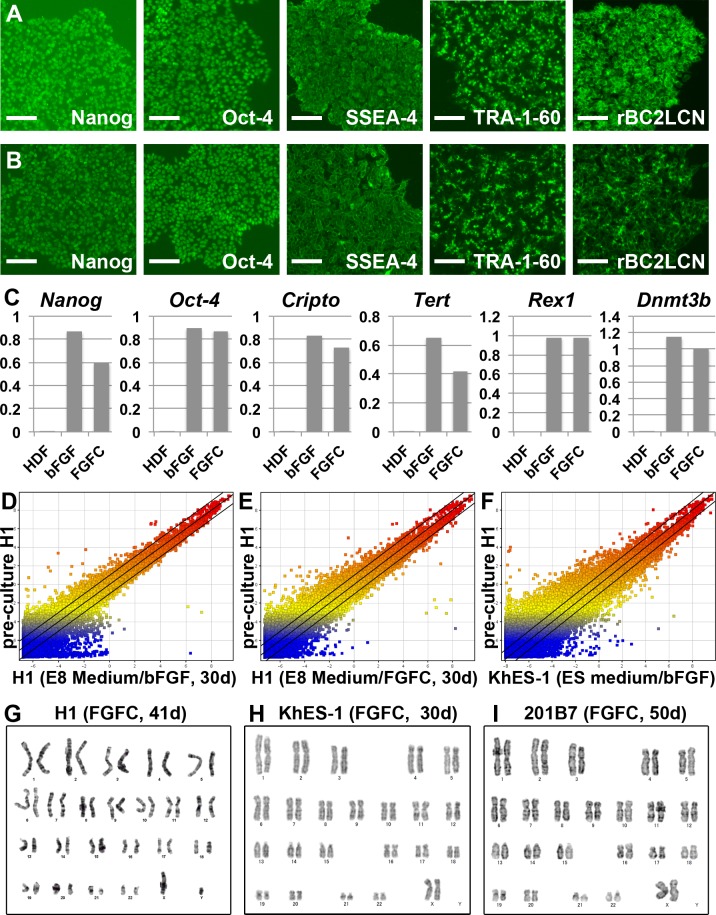
Replacement of bFGF by FGFC in long-term culture. (A-C) Pluripotent marker expression in H1 ESCs maintained with E8 Medium containing bFGF (A) or FGFC (B) for 30 days through 10 passages. The results of a single independent experiment are shown. (C) The expression levels of pluripotent marker genes (*Nanog*, *Oct-4*, *Cripto*, *Tert*, *Rex1* and *Dnmt3b*) were analyzed by quantitative RT-PCR. The graphs represent relative gene expression levels when the level of H1 ESCs prior to starting the series of cultures was set as 1. Human dermal fibroblasts (HDFs) were used as a negative control. (D-F) Scatter plots showing log_2_ transformed average expression values from gene expression profiles between pre-culture H1 ESCs and H1 ESCs cultured with bFGF (D, corresponding to the sample in A) or FGFC (E, corresponding to the sample in B) for 30 days (30d) or KhES-1 ESCs maintained with bFGF (F) using arrayed 60 k probe sets. A typical data set from duplicate microarray experiments is shown. The full array data set was deposited in the GEO databank (GSE55428). Black lines indicate 2-fold up-regulation or down-regulation. (G-I) G-banded karyotype analyses. The results of each single culture experiment are shown. (G) H1 ESCs, which had been cultured in MEF-conditioned medium with FGFC for 41 days (41d) through 10 passages (46,XY[20]). (H) KhES-1 ESCs, which had been cultured in medium with FGFC, instead of bFGF, on MMC-MEF feeder cells for 30 days (30d) through 10 passages (46,XX[20]). (I) 201B7 iPSCs, which had been cultured in medium with FGFC, instead of bFGF, on MMC-MEF feeder cells for 50 days (50d) through 10 passages (46,XX[20]). The karyotype notation complies with ISCN. Scale bar: 100 μm in (A and B).

We next evaluated the effects of long-term culture in FGFC-containing medium on hESCs (Fig 3). H1 ESCs were cultured with either bFGF or FGFC for 30 days through 10 passages. The expression levels of the pluripotency markers were not significantly different between the two conditions (Fig 3A–3C). Furthermore, the global gene expression profiles of H1 hESCs after culture with bFGF or FGFC for 30 days were very similar to their expression profiles before culture (Fig 3D and 3E). These expression profiles exhibited more even scatter plots than the scatter plot between pre-culture cells and the KhES-1 cell line (Fig 3F), which served as a benchmark for pluripotency. Taken together, these results indicate that replacement of bFGF with FGFC maintained the pluripotency of hESCs.

The karyotype of H1 ESCs was also analyzed after long-term culture with FGFC (Fig 3G, S1A–S1D and S2A Figs). Importantly, the karyotypes of these cells remained unchanged (46,XY[20]). Similar results were observed in both KhES-1 hESCs (Fig 3H, S1E–S1H and S2B Figs) and in 201B7 hiPSCs (Fig 3I, S1I–S1L and S2C Figs) grown on a feeder layer of MEFs.

Next, we analyzed the subtle differences in the gene expression levels in cells cultured with bFGF or FGFC in long-term culture (S2, S3, S4, S5, S6, S7, S8, S9, S10 and S11 Tables). Of 42,405 DNA microarray probes, 2,912 probes in H1 hESCs cultured with E8 Medium (S2 and S3 Tables), 1,866 probes in H1 hESCs cultured with MEF-conditioned medium (S4 and S5 Tables), 1,346 probes in KhES-1 hESCs (S6 and S7 Tables), 2,611 probes in 201B7 hiPSCs (S8 and S9 Tables), and 3,120 probes in 253G1 hiPSCs (S10 and S11 Tables) showed significant differences (more than 2.0-fold change in expression) when cultured in FGFC-containing medium compared with culture in bFGF-containing medium. There was no probe that showed a common significant difference for all five culture conditions (S2, S3, S4, S5, S6, S7, S8, S9, S10 and S11 Tables). Furthermore, no common gene ontology class was extracted from all probe lists. These results indicated that there were no common changes induced by replacement of bFGF with FGFC in human pluripotent stem cells. In addition, we analyzed the gene expression levels of early differentiation markers for three germ lineages (S3 Fig). The expression levels of these genes were similar in human pluripotent stem cells cultured with bFGF or FGFC.

**Fig 4:**
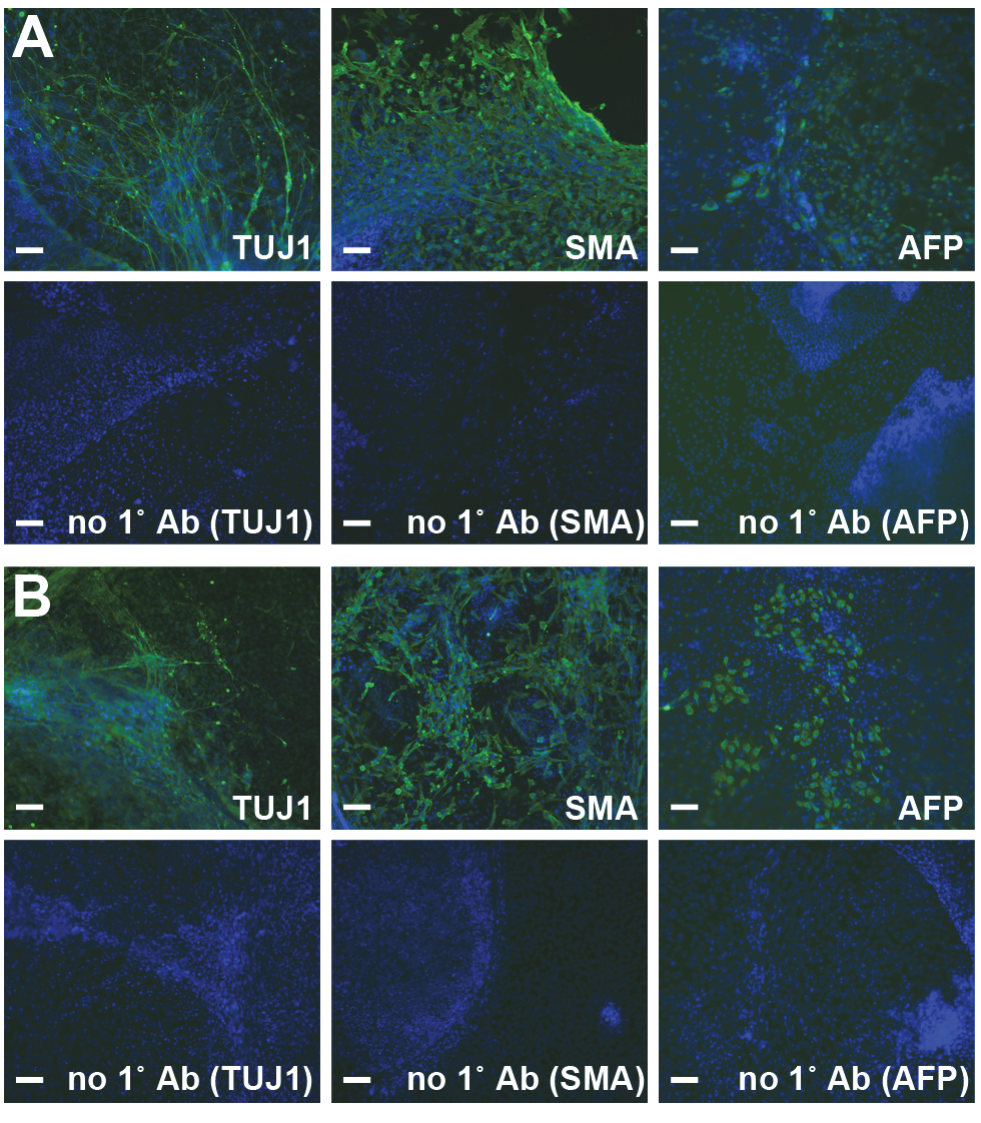
FGFC maintained the pluripotency of human ESCs in long-term culture. hESCs were first maintained in E8 Medium with either bFGF (A) or FGFC (B) and then prepared for the *in vitro* differentiation assay. Differentiation into three germ lineages (neural, muscle, and liver) was detected by immunocytochemistry. The result of a single experiment is shown. TUJ1: anti-Class IIIβ-Tubulin, SMA: anti-human smooth muscle actin, AFP: anti-α-fetoprotein. no 1° Ab: negative control cells stained with each secondary antibody alone. Scale bar: 100 μm.

Finally, we also assessed whether H1 ESCs grown in the long-term culture using FGFC maintained pluripotency to differentiate into all three germ lineages. H1 ESCs were induced to spontaneously differentiate by EB formation *in vitro* (Fig 4). Expression of the neural marker TUJ1, SMA, and anti-α-fetoprotein was investigated and observed in cells derived from hESCs after a 30-day culture with FGFC (Fig 4B) or bFGF (Fig 4A). These markers represented all three germ lineages (ectoderm, mesoderm, and endoderm) of the embryo, therefore suggesting that FGFC was capable of replacing bFGF in culture media used for hESCs.

In summary, we have shown that FGFC is an effective tool for the stable maintenance of human pluripotent stem cells and is able to replace bFGF in ordinary culture medium. Production of recombinant FGFC protein can be carried out in a prokaryotic expression system, such as *E*. *coli* cells, and may therefore be more easy and cost-effective than production of bFGF. Furthermore, owing to the superiority of FGFC over bFGF in terms of stability against heat and proteolytic digestion during storage [11], FGFC may prove to be a better FGF supplement in more demanding culture settings, such as those used for regenerative medicine. Our results, which showed the effects of FGFC on human pluripotent stem cells in culture, may be applicable to other types of stem cells that require FGF signaling, such as neural stem cells, where FGFC may also be used as a replacement for FGF in culture media.

## Supporting Information

S1 Fig Replacement of bFGF by FGFC maintained pluripotency markers and global gene expression in human ESCs in long-term culture. (A) Expression of pluripotency markers in H1 ESCs cultured in FGFC-containing MEF-conditioned medium (MEF-CM) for 41 days (41d) through 10 passages. (B–D) Scatter plots using arrayed 60 k probe sets, showing log_2_ transformed average expression values from gene expression profiles between pre-culture H1 ESCs and H1 ESCs cultured with bFGF (B) or with FGFC (C, corresponding to the sample in (A)), or H1 ESCs maintained in E8 Medium with bFGF (D). (E) Expression of pluripotency markers in KhES-1 ESCs cultured in FGFC-containing medium on MMC-MEF feeder cells for 30 days (30d) through 10 passages. (F–H) Scatter plots using arrayed 60 k probe sets, showing log_2_ transformed average expression values from gene expression profiles between pre-culture KhES-1 ESCs and KhES-1 ESCs cultured with bFGF (F) or with FGFC (G, corresponding to the sample in (E)), or H1 ESCs maintained in E8 Medium with bFGF (H). (I) Pluripotent marker expression of 201B7 iPSCs cultured in FGFC-containing medium on MMC-MEF feeder cells for 50 days (50d) through 10 passages. (J–L) Scatter plots using arrayed 60 k probe sets, showing log_2_ transformed average expression values from gene expression profiles between pre-culture 201B7 iPSCs and 201B7 iPSCs cultured with bFGF (J) or FGFC (K, corresponding to the sample in (I)), or KhES-1 ESCs maintained with bFGF (L). (M–O) 253G1 iPSCs cultured with E8 Medium containing bFGF or FGFC for 36 days (36d) through 9 passages. Scatter plots using arrayed 60 k probe sets, showing log_2_ transformed average expression values from gene expression profiles between pre-culture 253G1 iPSCs and 253G1 iPSCs cultured with bFGF (M) or FGFC (N) or 201B7 iPSCs maintained with bFGF (O). The full array data set was deposited in the GEO databank (GSE55428). Black lines indicate 2-fold up-regulation or down-regulation. Typical data sets of duplicate microarray experiments for each single experiment are shown. Scale bar: 100 μm in (A), (E), and (I).
(TIF)

S2 Fig Quantitative RT-PCR analyses of the expression of pluripotent marker genes. The graphs represent the relative gene expression levels when the level in cells prior to starting the series of cultures was set as 1. Human dermal fibroblasts (HDFs) were used as a negative control. (A) H1 ESCs cultured in bFGF- or FGFC-containing MEF-conditioned medium (MEF-CM) for 41 days through 10 passages. (B) KhES-1 ESCs cultured in FGFC-containing medium on MMC-MEF feeder cells for 30 days through 10 passages. (C) 201B7 iPSCs cultured in bFGF- or FGFC-containing medium on MMC-MEF feeder cells for 50 days through 10 passages. (D) 253G1 iPSCs cultured in E8 Medium containing bFGF or FGFC on Matrigel for 36 days through 9 passages. All pluripotent marker genes were steadily expressed during culture with bFGF or FGFC.
(TIF)

S3 Fig Heat map showing the expression of differentiation marker genes expression. Data for the expression of 14 differentiation marker genes are shown from parts of the DNA microarray data in Fig 3 (H1 ESCs in E8 Medium system) and Fig. S1 (H1 ESCs in MEF-conditioned medium [MEF-CM], KhES-1 on MMC-MEF feeder cells, 201B7 on MMC-MEF feeder cells, and 253G1 in E8 Medium system). As a positive control, H1 ESCs, which had been maintained with E8 Medium, were supplied for *in vitro* differentiation assays via EB formation (H1 [differentiated]). The full array data set was deposited in the GEO databank (GSE55428). The color of each square indicates the relative expression level of the gene indicated on the side; higher expression levels are indicated in red, and lower expression levels are indicated in blue. Gene symbols and specific linages as follows: PDGFR-β: platelet-derived growth factor receptor, beta polypeptide (mesoderm); TNNT2: troponin T type 2 (heart); TH: tyrosine hydroxylase (neuron); SOX17: SRY (sex determining region Y)-box 17 (endoderm); MYH6: myosin, heavy chain 6, cardiac muscle, alpha (heart); PPAR-γ: peroxisome proliferator-activated receptor gamma (fat); CDX2: caudal type homeobox 2 (intestine); ZIC1: Zic family member 1 (ectoderm and mesoderm); SOX1: SRY (sex determining region Y)-box 1 (ectoderm); KRT14: keratin 14 (skin); PAX6: paired box 6 (ectoderm); ALB: albumin (liver); SOX7: SRY (sex determining region Y)-box 7 (endoderm); T: brachyury homolog, beta polypeptide (mesoderm and endoderm).
(TIF)

S1 Table Proteome Profiler Human Phospho-MAPK Array coordinates (modified from the protocol of R&D Systems, Inc). (XLSX)

S2 Table List of 708 probes exhibiting increased expression (by 2-fold or more) under FGFC culture conditions compared with bFGF culture conditions. H1 ESCs were cultured in E8 medium with either bFGF or FGFC for 30 days through 10 passages. The full array data set was deposited in the GEO databank (GSE55428).
(XLSX)

S3 Table List of 2,204 probes exhibiting reduced expression (by 2-fold or more) under FGFC culture conditions compared with bFGF culture conditions. H1 ESCs were cultured in E8 medium with either bFGF or FGFC for 30 days through 10 passages. The full array data set was deposited in the GEO databank (GSE55428).
(XLSX)

S4 Table List of 603 probes exhibiting increased expression (by 2-fold or more) under FGFC culture conditions compared with bFGF culture conditions. H1 ESCs were cultured in MEF-conditioned medium with bFGF or FGFC for 41 days through 10 passages. The full array data set was deposited in the GEO databank (GSE55428).
(XLSX)

S5 Table List of 1,263 probes exhibiting reduced expression (by 2-fold or more) under FGFC culture conditions compared with bFGF culture conditions. H1 ESCs were cultured in MEF-conditioned medium with bFGF or FGFC for 41 days through 10 passages. The full array data set was deposited in the GEO databank (GSE55428).
(XLSX)

S6 Table List of 785 probes exhibiting increased expression (by 2-fold or more) under FGFC culture conditions compared with bFGF culture conditions. KhES-1 ESCs were cultured in either bFGF- or FGFC-containing medium on MMC-MEF feeder cells for 30 days through 10 passages. The full array data set was deposited in the GEO databank (GSE55428).
(XLSX)

S7 Table List of 561 probes exhibiting reduced expression (by 2-fold or more) under FGFC culture conditions compared with bFGF culture conditions. KhES-1 ESCs were cultured in either bFGF- or FGFC-containing medium on MMC-MEF feeder cells for 30 days through 10 passages. The full array data set was deposited in the GEO databank (GSE55428).
(XLSX)

S8 Table List of 490 probes exhibiting increased expression (by 2-fold or more) under FGFC culture conditions compared with bFGF culture conditions. 201B7 iPSCs were cultured in either bFGF- or FGFC-containing medium on MMC-MEF feeder cells for 50 days through 10 passages. The full array data set was deposited in the GEO databank (GSE55428).
(XLSX)

S9 Table List of 2,121 exhibiting reduced expression (by 2-fold or more) under FGFC culture conditions compared with bFGF culture conditions. 201B7 iPSCs were cultured in either bFGF- or FGFC-containing medium on MMC-MEF feeder cells for 50 days through 10 passages. The full array data set was deposited in the GEO databank (GSE55428).
(XLSX)

S10 Table List of 1,001 probes exhibiting increased expression (by 2-fold or more) under FGFC culture conditions compared with bFGF culture conditions. 253G1 iPSCs were cultured in E8 Medium with either bFGF or FGFC for 36 days through 9 passages. The full array data set was deposited in the GEO databank (GSE55428).
(XLSX)

S11 Table List of 2,119 probes exhibiting reduced expression (by 2-fold or more) under FGFC culture conditions compared with bFGF culture conditions. 253G1 iPSCs were cultured in E8 Medium with either bFGF or FGFC for 36 days through 9 passages. The full array data set was deposited in the GEO databank (GSE55428).
(XLSX)
