# Supplementary figures and images for: A Stable Chimeric Fibroblast Growth Factor (FGF) Can Successfully Replace Basic FGF in Human Pluripotent Stem Cell Culture
Source: PLoS One. 2015 Apr 7;10(4):e0118931. doi: 10.1371/journal.pone.0118931 (PMC4388338; doi:10.1371/journal.pone.0118931)

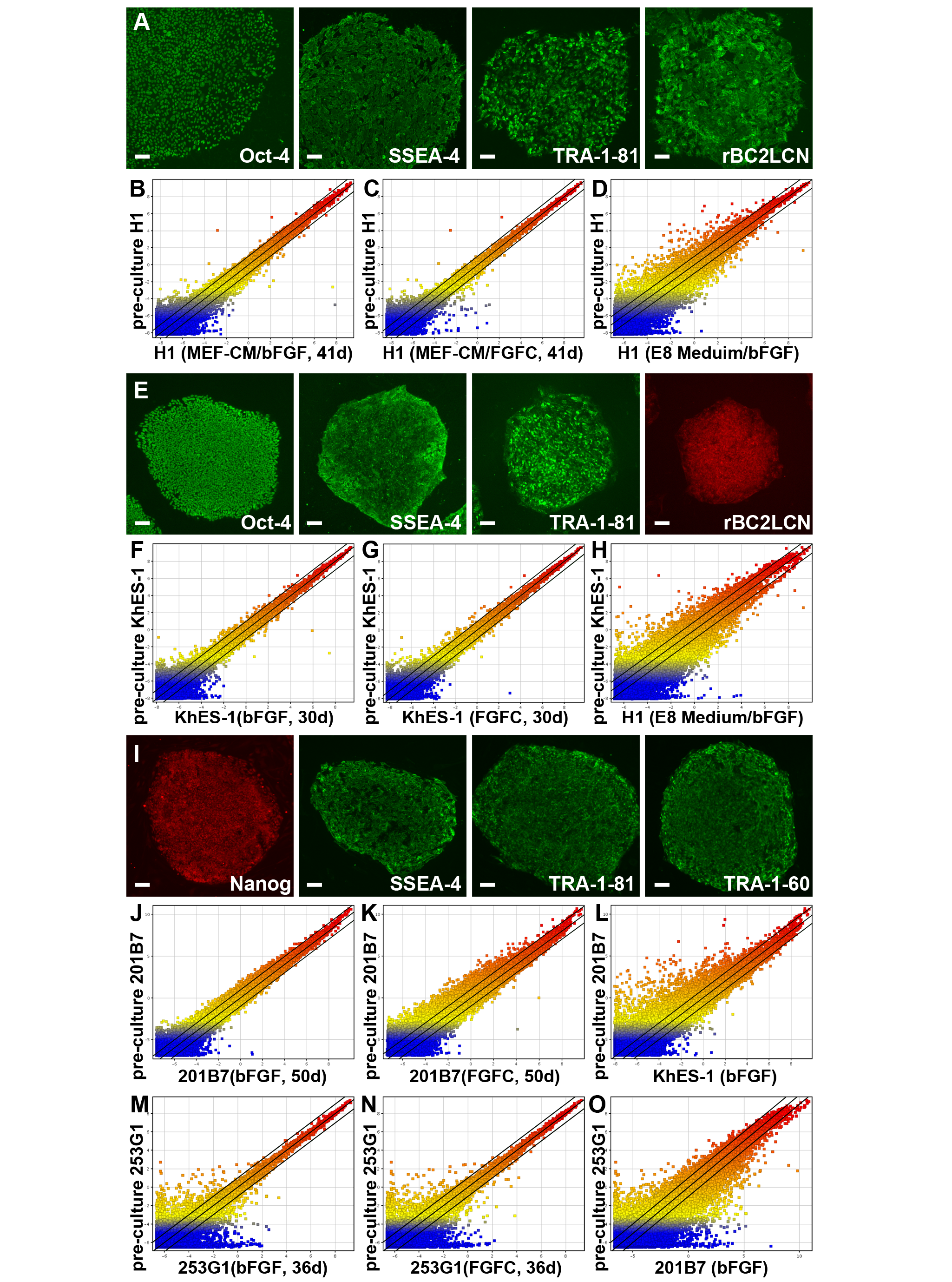

Supplement: S1 Fig — (A) Expression of pluripotency markers in H1 ESCs cultured in FGFC-containing MEF-conditioned medium (MEF-CM) for 41 days (41d) through 10 passages. (B–D) Scatter plots using arrayed 60 k probe sets, showing log2 transformed average expression values from gene expression profiles between pre-culture H1 ESCs and H1 ESCs cultured with bFGF (B) or with FGFC (C, corresponding to the sample in (A)), or H1 ESCs maintained in E8 Medium with bFGF (D). (E) Expression of pluripotency markers in KhES-1 ESCs cultured in FGFC-containing medium on MMC-MEF feeder cells for 30 days (30d) through 10 passages. (F–H) Scatter plots using arrayed 60 k probe sets, showing log2 transformed average expression values from gene expression profiles between pre-culture KhES-1 ESCs and KhES-1 ESCs cultured with bFGF (F) or with FGFC (G, corresponding to the sample in (E)), or H1 ESCs maintained in E8 Medium with bFGF (H). (I) Pluripotent marker expression of 201B7 iPSCs cultured in FGFC-containing medium on MMC-MEF feeder cells for 50 days (50d) through 10 passages. (J–L) Scatter plots using arrayed 60 k probe sets, showing log2 transformed average expression values from gene expression profiles between pre-culture 201B7 iPSCs and 201B7 iPSCs cultured with bFGF (J) or FGFC (K, corresponding to the sample in (I)), or KhES-1 ESCs maintained with bFGF (L). (M–O) 253G1 iPSCs cultured with E8 Medium containing bFGF or FGFC for 36 days (36d) through 9 passages. Scatter plots using arrayed 60 k probe sets, showing log2 transformed average expression values from gene expression profiles between pre-culture 253G1 iPSCs and 253G1 iPSCs cultured with bFGF (M) or FGFC (N) or 201B7 iPSCs maintained with bFGF (O). The full array data set was deposited in the GEO databank (GSE55428). Black lines indicate 2-fold up-regulation or down-regulation. Typical data sets of duplicate microarray experiments for each single experiment are shown. Scale bar: 100 μm in (A), (E), and (I). (TIF) [file pone.0118931.s001.tif]

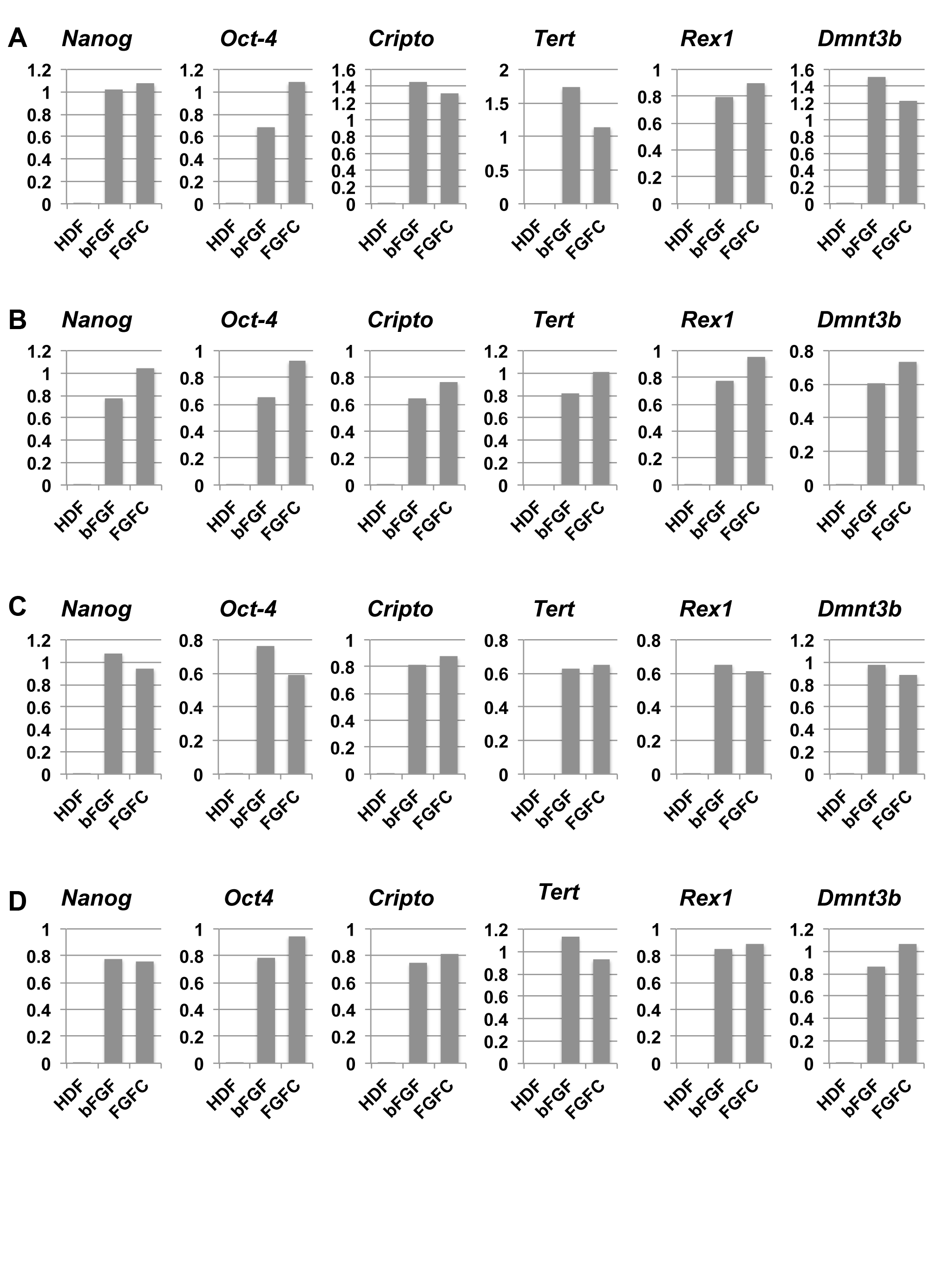

Supplement: S2 Fig — The graphs represent the relative gene expression levels when the level in cells prior to starting the series of cultures was set as 1. Human dermal fibroblasts (HDFs) were used as a negative control. (A) H1 ESCs cultured in bFGF- or FGFC-containing MEF-conditioned medium (MEF-CM) for 41 days through 10 passages. (B) KhES-1 ESCs cultured in FGFC-containing medium on MMC-MEF feeder cells for 30 days through 10 passages. (C) 201B7 iPSCs cultured in bFGF- or FGFC-containing medium on MMC-MEF feeder cells for 50 days through 10 passages. (D) 253G1 iPSCs cultured in E8 Medium containing bFGF or FGFC on Matrigel for 36 days through 9 passages. All pluripotent marker genes were steadily expressed during culture with bFGF or FGFC. (TIF) [file pone.0118931.s002.tif]

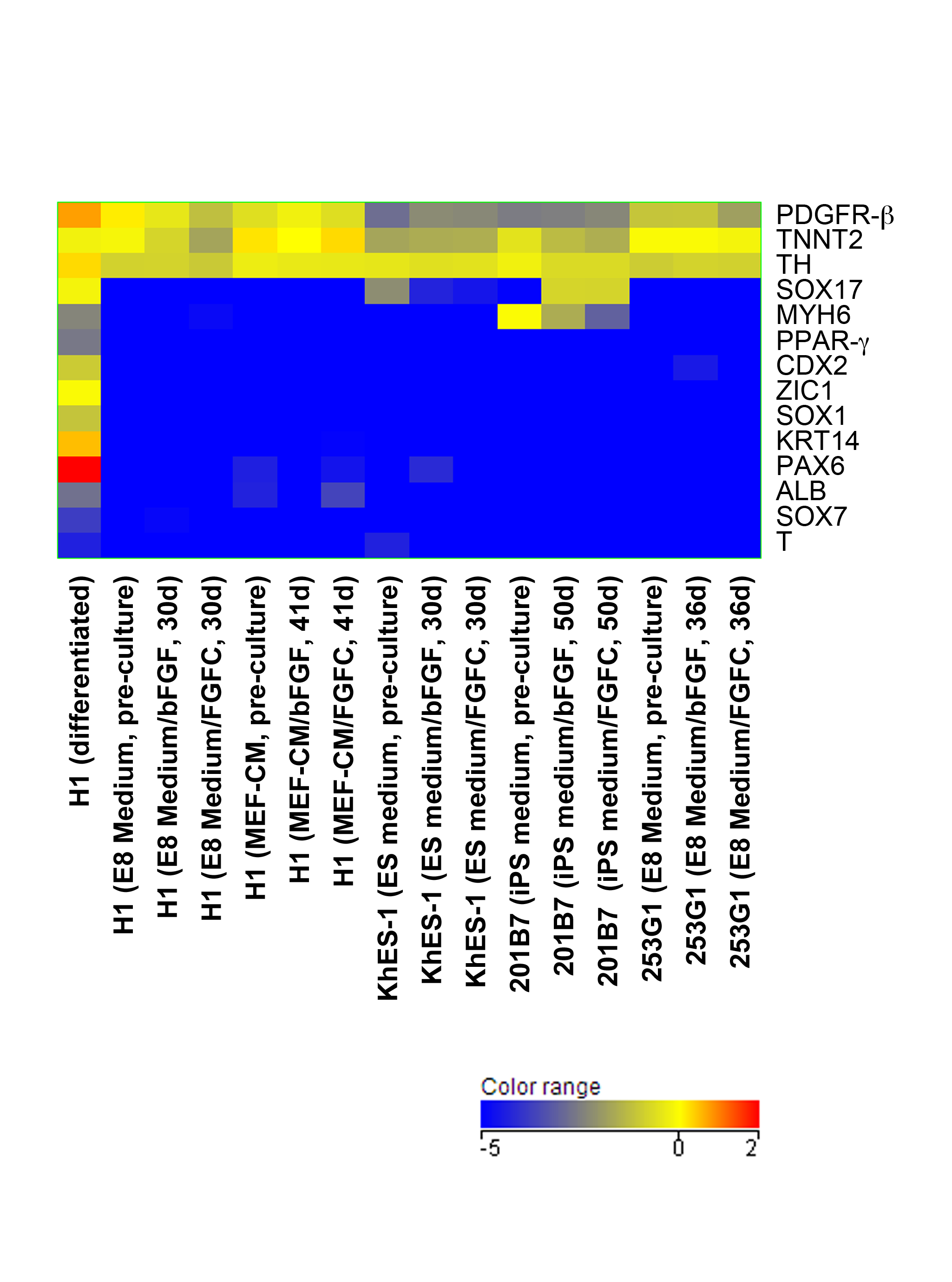

Supplement: S3 Fig — Data for the expression of 14 differentiation marker genes are shown from parts of the DNA microarray data in Fig 3 (H1 ESCs in E8 Medium system) and Fig. S1 (H1 ESCs in MEF-conditioned medium [MEF-CM], KhES-1 on MMC-MEF feeder cells, 201B7 on MMC-MEF feeder cells, and 253G1 in E8 Medium system). As a positive control, H1 ESCs, which had been maintained with E8 Medium, were supplied for in vitro differentiation assays via EB formation (H1 [differentiated]). The full array data set was deposited in the GEO databank (GSE55428). The color of each square indicates the relative expression level of the gene indicated on the side; higher expression levels are indicated in red, and lower expression levels are indicated in blue. Gene symbols and specific linages as follows: PDGFR-β: platelet-derived growth factor receptor, beta polypeptide (mesoderm); TNNT2: troponin T type 2 (heart); TH: tyrosine hydroxylase (neuron); SOX17: SRY (sex determining region Y)-box 17 (endoderm); MYH6: myosin, heavy chain 6, cardiac muscle, alpha (heart); PPAR-γ: peroxisome proliferator-activated receptor gamma (fat); CDX2: caudal type homeobox 2 (intestine); ZIC1: Zic family member 1 (ectoderm and mesoderm); SOX1: SRY (sex determining region Y)-box 1 (ectoderm); KRT14: keratin 14 (skin); PAX6: paired box 6 (ectoderm); ALB: albumin (liver); SOX7: SRY (sex determining region Y)-box 7 (endoderm); T: brachyury homolog, beta polypeptide (mesoderm and endoderm). (TIF) [file pone.0118931.s003.tif]
